# Association of lipid parameters with the development of disease complications in patients with limited cutaneous systemic sclerosis: a prospective exploratory cohort study

**DOI:** 10.3389/fnut.2026.1794319

**Published:** 2026-03-24

**Authors:** Leyla Schweiger, Andreas Meinitzer, Heimo Strohmaier, Günther Silbernagel, Florentine Moazedi-Fürst, Viktoria Nemecz, Katharina Kurzmann-Gütl, Marianne Brodmann, Franz Hafner, Philipp Jud

**Affiliations:** 1 Division of Angiology, Department of Internal Medicine, Medical University of Graz, Graz, Austria; 2 Institute of Medical and Chemical Laboratory Diagnostics, Medical University Graz, Graz, Austria; 3 Center of Medical Research (ZMF), Medical University of Graz, Graz, Austria; 4 Division of Rheumatology, Department of Internal Medicine, Medical University of Graz, Graz, Austria

**Keywords:** cardiovascular risk, endothelial dysfunction, limited cutaneous systemic sclerosis, lipoprotein metabolism, vascular biomarkers

## Abstract

**Background:**

Dyslipidemia may contribute to pathogenesis and disease complications in limited cutaneous systemic sclerosis (lcSSc), while data about its associations with endothelial dysfunction and clinical lcSSc-specific parameters as well as its predictive role on disease complications are limited. This study investigated the relationship between lipid parameters, endothelial dysfunction, and disease complications in patients with lcSSc.

**Methods:**

38 patients with lcSSc and 38 matched controls with primary Raynaud’s phenomenon were analyzed at baseline regarding lipid parameters, parameters of endothelial dysfunction and clinical lcSSc-specific parameters. During a subsequent 3-year follow-up period, patients with lcSSc were prospectively observed for disease complications.

**Results:**

Patients with lcSSc exhibited lower high-density lipoprotein (HDL) levels (*p* = 0.032) and total HDL particles (*p* = 0.001) as well as higher triglyceride levels (*p* = 0.018), triglycerides/HDL ratio (*p* = 0.013) and atherogenic index (*p* = 0.012) compared to controls. Large low-density lipoprotein (LDL) particles correlated positively with endothelial microparticles (*r* = 0.464, *p* = 0.044), while very low-density lipoprotein size correlated negatively with symmetric dimethylarginine (*r* = −0.480, *p* = 0.033). No significant correlations were observed between lipid parameters and clinical lcSSc-specific parameters. Incidence of macrovascular events and interstitial lung disease were associated with a few selected lipid parameters (all *p* < 0.05).

**Conclusion:**

Patients with lcSSc showed a more pro-atherogenic lipid profile. Although statistically significant, these results were of modest magnitude and should be interpreted in the context of the exploratory design. Associations with endothelial dysfunction and clinical disease parameters were minimal or non-significant. Selected lipid parameters may be associated with disease complications in lcSSc, but these findings require confirmation in larger, adequately powered studies due to the small sample size and exploratory analyses.

## 1 Introduction

Systemic sclerosis (SSc) is a complex autoimmune connective tissue disorder characterized by microvasculopathy, immune dysregulation, and progressive fibrosis (1, 2). Among its subtypes, limited cutaneous systemic sclerosis (lcSSc) presents with distinct clinical and serological features, yet the mechanisms underlying disease complications remain incompletely understood (3). Recent evidence highlights the role of lipid metabolism in modulating vascular and fibrotic processes central to SSc pathogenesis. Alterations in classic lipid parameters, including total cholesterol (TC), high-density lipoprotein (HDL), low-density lipoprotein (LDL), and triglycerides have been observed in SSc populations (2, 4). Previous reports, including meta-analysis, indicate that patients with lcSSc often demonstrate significantly reduced HDL and elevated triglyceride levels, with less consistent changes in TC compared to healthy controls. These changes are thought to promote a shift toward a pro-atherogenic lipid profile, thus potentially increasing the risk of both microvascular and macrovascular complications (2, 3). Furthermore, mechanistic studies implicate also qualitative lipid changes, like an impaired HDL cholesterol efflux capacity in SSc, which correlates with skin thickness (5). Elevated LDL and triglyceride levels have also been associated with increased risk of interstitial lung disease (ILD) in SSc, possibly by mechanisms involving oxidative stress and immune activation, which may lead to more severe tissue fibrosis (6–8). Moreover, distinctive changes in sphingomyelin and ceramide species have been described in SSc, supporting an atherogenic profile (9). Collectively, these findings suggest that especially abnormalities of HDL, LDL, and triglycerides are intricately associated with disease complications in lcSSc.

Although previous studies have documented alterations of lipid parameters in SSc, suggesting a pro-atherogenic lipid profile that may contribute to disease complications, the strength and directionality of associations between specific lipid subparameter and lipid fractions as well as clinical outcomes in lcSSc remain incompletely understood. Most available data have focused on conventional lipid parameters leading to uncertainty about the specific roles of distinct lipid subfractions, like HDL or LDL particles (2–4). Other conventional lipid parameters like intermediate-density lipoprotein (IDL) or very low-density lipoprotein (VLDL), have been less investigated, especially in lcSSc. Furthermore, potential associations between lipids and parameters of endothelial dysfunction have not been investigated in lcSSc to the best of our knowledge. Moreover, the predictive value of lipid parameters on disease complications of lcSSc needs to be yet elucidated.

Thus, this study aims to evaluate which lipid parameters are altered in lcSSc compared to controls by measuring lipid parameters with nuclear magnetic resonance (NMR) method. Thereby, the relationship between lipids with endothelial dysfunction markers and lcSSc-specific clinical features was assessed and the predictive role of lipid parameters for disease complications in lcSSc was evaluated.

## 2 Materials and methods

### 2.1 Study design and patient cohort

This investigation represents a predefined exploratory sub-study derived from previously established cohorts investigating endothelial dysfunction and clinical outcomes in lcSSc and parts of this cohort have been analyzed and reported previously (10–12). The present analysis utilizes the same patient population but investigates retrospectively measured NMR-based lipid parameters addressing a distinct research hypothesis. The present study links *post-hoc* baseline NMR-based lipid measurements with endothelial dysfunction and clinical parameters as well as with vascular and clinical events in lcSSc. The initial baseline study evaluated endothelial dysfunction in patients with lcSSc who had not yet developed vasculopathy-related complications. It comprised 38 individuals with confirmed lcSSc and 38 control subjects diagnosed with primary Raynaud’s phenomenon, matched 1:1 for age, sex, and race (10). Primary Raynaud’s phenomenon was chosen as a control group because these individuals exhibit vasospastic symptoms similar to patients with lcSSc but lack autoimmune, fibrotic, or microvascular structural changes. This allows for better separation of disease-specific alterations from nonspecific effects related to vasospasm itself. Previous studies have also used patients with primary Raynaud’s phenomenon as an appropriate control group in microvascular and lipid research due to its absence of systemic involvement (13, 14). The subsequent follow-up study prospectively examined the incidence of disease-specific complications and cardiovascular events among 38 patients with lcSSc over a 3-year observational period, while five patients were lost to follow-up. Owing to the original study design, control subjects were not included in the follow-up visits (11). Detailed descriptions of the study protocols have been published previously (10, 11). The relationship between the previously published analyses and the present lipid-focused sub-study is transparently disclosed to ensure methodological clarity. At baseline, lipid parameters, markers of endothelial dysfunction, and lcSSc-specific clinical data were collected. During annual follow-up visits, the occurrence of lcSSc-related disease complications and cardiovascular outcomes defined as vascular and clinical events were documented throughout the observation period.

Eligibility for inclusion in the lcSSc group required a diagnosis based on the current EULAR/ACR classification criteria (15). Exclusion criteria at enrolment comprised an age below 18 years, the presence of diffuse cutaneous SSc or other connective tissue disorders, current or previous pulmonary arterial hypertension (PAH), digital ulcers (DU), endoscopically verified reflux, diabetes mellitus, and symptomatic atherosclerotic cardiovascular disease. Additional exclusions included recent pregnancy, active malignancy, acute infection at the time of enrolment, and intake of vasoactive agents (prostanoids, calcium channel blockers, phosphodiesterase-5 inhibitors, or endothelin receptor antagonists) within 24 h prior to study participation.

The primary endpoint of the present analysis was the comparison of lipid parameters between patients with lcSSc and controls with primary Raynaud’s phenomenon at baseline. Secondary endpoints included correlations between lipid parameters, lcSSc-specific parameters, and parameters of endothelial dysfunction at baseline, as well as associations between lipid parameters and development of vascular and clinical events.

### 2.2 Biochemical analyses

Fasting blood samples were collected from each participant at study inclusion to assess lipid parameters, comprising TC, HDL, IDL, LDL, VLDL, triglycerides, size of HDL, LDL, and VLDL, as well as HDL, LDL, and VLDL particles. Furthermore, von Willebrand factor (vWF) antigen (vWF: Ag) and activity (vWF: Ac), asymmetric dimethylarginine (ADMA), symmetric dimethylarginine (SDMA), homoarginine, arginine, and CD31+/CD42b− endothelial microparticles (EMP) were measured as laboratory biomarkers of endothelial dysfunction from fasting blood samples at baseline. TC, HDL, LDL, triglycerides, vWF: Ag and vWF: Ac were measured by routine laboratory work-up. Concentrations of VLDL, IDL, the size and particle counts of HDL, LDL and VLDL were determined by NMR using the AXINON lipoFIT platform at Numares AG (Am Biopark, Regensburg, Germany) from frozen blood samples (−80 °C) *post-hoc*. This analysis utilized an Avance III HD nuclear magnetic resonance spectrometer, an Ascend 600 MHz magnet (Bruker), TopSpin 3.2 (Bruker) and Axinon Suite V.1.0.0.1 (Numares) software (16, 17). Triglycerides/HDL along with the atherogenic coefficient and atherogenic index, were also calculated *post-hoc*, given their established association with cardiovascular disease (18, 19). The inclusion of this extensive NMR-based lipoprotein panel was motivated by previous evidence suggesting that particle number and size may provide additional insights beyond conventional lipid measures, possibly capturing early vascular or fibrotic risk relevant in lcSSc. Despite the small sample size, this panel was used in an exploratory manner to identify candidate lipid patterns warranting further investigation. Adjustments for confounders such as age, body-mass-index, disease duration, and medication use were limited due to the small sample size and limited events, to avoid overfitting and unstable models. Descriptive statistics and effect sizes were reported to aid interpretation. Sensitivity analyses adjusting for age did not materially alter associations (data not shown). Parameters of the arginine metabolism were assessed by high-performance liquid chromatography while EMP were measured according to the recommendations published by Meinitzer et al. (20) and Cossarizza et al. (21). Detailed information about the biochemical measurements of the respective parameters of endothelial dysfunction have been previously published (10).

### 2.3 LcSSc-specific parameters

LcSSc-specific parameters included capillaroscopic skin ulcer risk index (CSURI), microangiopathy evolution score (MES), modified Rodnan Skin Score (mRSS), UCLA SCTC GIT 2.0 total and constipation score, DETECT score, and EUSTAR index. Nailfold videocapillaroscopy from on the second to fifth fingers of both hands (Skinview, Optometron Ltd., Oskar-Messterstr., Ismaning, Germany) were used for assessing CSURI and MES (22, 23). mRSS was obtained by standardized physical examination (24). The UCLA SCTC GIT 2.0 total and constipation scores were derived from validated questionnaires, while DETECT score and EUSTAR index were calculated in accordance with previously published methodologies (25–27).

### 2.4 Vascular assessment

Functional indices of endothelial dysfunction, including flow-mediated dilation, nitroglycerin-mediated dilation, pulse wave velocity, and augmentation index, were evaluated at baseline. Comprehensive methodological details regarding these assessments have been reported previously (10). In summary, flow-mediated dilation and nitroglycerin-mediated dilation were assessed following the guidelines outlined by Corretti et al. (28), whereas pulse wave velocity and augmentation index were measured using an oscillometric device (I.E.M. Mobil-O-Graph, I.E.M., Stolberg, Germany) with automated pulse wave analysis.

### 2.5 Vascular and clinical events

Developments of new DU and PAH during follow-up were defined as microvascular events, and macrovascular events were defined as development of new symptomatic coronary heart disease, carotid and vertebral artery disease, and peripheral artery disease. Clinical events were defined as all micro- and macrovascular events including also the development of ILD, renal crisis and esophageal dysfunction during the follow-up. Further details about definitions and recording of respective vascular and clinical events have been previously published (11).

### 2.6 Statistical analysis

Kolmogorov–Smirnov test was applied to assess the normality of data distribution. Normal distributed data were presented as means with standard deviations (SD), and non-normally distributed data as medians with interquartile ranges. Group comparisons were performed using Student’s *t*-test for normally distributed variables and the Mann–Whitney U test for non-normally distributed variables. Correlations were evaluated using Pearson’s correlation coefficient for normally distributed variables and Spearman’s rank correlation coefficient for non-normally distributed variables. Associations between categorical and continuous variables were examined by the eta coefficient followed by one-way analysis of variance (ANOVA). Effect size of ANOVA was given by eta squared (*η*^2^). Given the exploratory nature of the study and the large number of statistical tests performed, correlation analyses and ANOVA were adjusted for multiple testing using the Holm–Bonferroni correction. Statistical significance was defined as an adjusted *p*-value <0.05. All analyses were conducted using SPSS software, version 29.0 (IBM Corp., Armonk, NY, USA). Given the exploratory design and limited sample size, these adjusted analyses were not intended for confirmatory inference but rather to reduce the likelihood of spurious findings while preserving descriptive interpretation of effect estimates.

### 2.7 Ethics statement

This study was approved by the local ethics committee (protocol number EK 29-361 ex 16/17) and was conducted in accordance with the ethical principles of the Declaration of Helsinki (1975, revised 2013). All participants received detailed information about the study procedures and provided written informed consent prior to inclusion.

## 3 Results

At baseline, the study enrolled 38 patients with confirmed lcSSc and 38 control subjects with primary Raynaud’s phenomenon. Twenty patients with lcSSc (52.6%) and twelve controls (31.6%) had a previously known hyperlipidemia (*p* = 0.103). Three patients with lcSSc (7.9%) and three controls (7.9%) had a therapy with statins each (*p* > 0.999). Patients with lcSSc were subsequently observed for three years. During the three-year observational period, 33 patients with lcSSc (86.8%) completed all follow-up visits, whereas five patients (13.2%) were lost to follow-up. Baseline patients’ characteristics are listed in Table 1. Results of endothelial dysfunction and lcSSc-specific parameters as well as follow-up patients’ characteristics, along with the occurrence of vascular and clinical events during the follow-up period, have been reported in detail previously (10–12).

**Table 1:** Baseline demographic and clinical characteristics of patients with lcSSc and controls.

Patient characteristics	lcSSc	Controls	*p*-value
Disease duration (years), mean ± SD	7.1 ± 5.8	5.7 ± 3.2	0.191
Sex, *n* (%)			
Male	2 (5.3)	2 (5.3)	>0.999
Female	36 (94.7)	36 (94.7)	>0.999
Age (years), mean (± SD)	57.9 ± 9.2	57.2 ± 9.0	0.622
BMI (kg/m^2^), mean (± SD)	23.61 ± 3.46	22.86 ± 3.53	0.487
Smoking, *n* (%)			
Active smokers	4 (10.5)	7 (18.4)	0.516
Ex-smokers	8 (21.1)	8 (21.1)	>0.999
Arterial hypertension, *n* (%)	14 (36.8)	12 (31.6)	0.809
Hyperlipidemia, *n* (%)	20 (52.6)	12 (31.6)	0.103
HbA1c (mmol/mol), median (25–75th percentile)	35 (34–37)	37 (34–38)	0.308
Malignancies, *n* (%)			
Recent	0 (0)	0 (0)	–
Prior	4 (10.5)	1 (2.6)	0.358
Prior digital ulcers, *n* (%)	0 (0)	0 (0)	–
Prior pulmonary arterial hypertension, *n* (%)	0 (0)	0 (0)	–
Prior interstitial lung disease, *n* (%)	2 (5.3)	0 (0)	0.493
Prior esophageal involvement, *n* (%)	9 (23.7)	0 (0)	**0.002**
Prior renal involvement, *n* (%)	2 (5.3)	0 (0)	0.493
Medication, *n* (%)			
ACE inhibitors/ARB	7 (18.4)	6 (15.8)	>0.999
Beta blockers	3 (7.9)	4 (10.5)	>0.999
Calcium channel blockers	6 (15.8)	5 (13.2)	>0.999
Diuretics	2 (5.3)	3 (7.9)	>0.999
Platelet aggregation inhibitors	6 (15.8)	4 (10.5)	0.736
Anticoagulants	3 (7.9)	0 (0)	0.240
Statins	3 (7.9)	3 (7.9)	>0.999
Immunosuppression, *n* (%)			
Cortisone	3 (7.9)	0 (0)	0.240
Methotrexate	1 (2.6)	0 (0)	>0.999
Mycophenolate mofetil	2 (5.3)	0 (0)	0.493
Rituximab	1 (2.6)	0 (0)	>0.999
Hydroxychloroquine	2 (5.3)	0 (0)	0.493
Abatacept	1 (2.6)	0 (0)	>0.999

BMI, body-mass index (kg/m^2^); HbA1c, glycated hemoglobin (mmol/mol); ACE, angiotensin-converting enzyme; ARB, angiotensin II receptor blocker. Data are presented as mean ± standard deviation, median (25th–75th percentile) or *n* (%), as appropriate. All significant values are indicated in bold.

### 3.1 Differences of lipid parameters between patients with lcSSc and controls

At baseline, patients with lcSSc demonstrated significantly lower HDL levels (*p* = 0.032) and lower total HDL particles compared to controls (*p* = 0.001). In contrast, triglyceride levels were significantly elevated (*p* = 0.018), accompanied by a higher triglycerides/HDL ratio and atherogenic index (*p* = 0.013, *p* = 0.012; respectively). Small LDL particles and the atherogenic coefficient showed higher, but non-significant values in lcSSc (*p* = 0.083; *p* = 0.096, respectively). No statistical differences were observed for the remaining lipid parameters (Table 2).

**Table 2:** Exploratory comparison of lipid parameters between patients with lcSSc and controls.

Lipid parameters	lcSSc	Controls	*p*-value
TC (mg/dL), median (25th–75th percentile)	215 (183–255)	209 (202–233)	0.640
HDL (mg/dL), mean (± SD)	72.5 (17.2)	81.7 (19.5)	**0.032**
IDL (mg/dL), mean (± SD)	61.5 (15.2)	58.5 (9.4)	0.320
LDL (mg/dL), mean (± SD)	122.9 (36.3)	116.0 (32.4)	0.385
VLDL (mg/dL), median (25th–75th percentile)	30.1 (24.6–34.4)	27.3 (25.5–31.4)	0.225
Triglycerides (mg/dL), median (25th–75th percentile)	95 (64–147)	67 (53–107)	**0.018**
HDL size (nm), mean (± SD)	9.4 (0.5)	9.5 (0.5)	0.294
LDL size (nm), median (25th–75th percentile)	21.6 (21.4–21.8)	21.6 (21.5–21.8)	0.660
VLDL size (nm), mean (± SD)	48.4 (2.6)	48.9 (2.7)	0.431
HDL particles (nmol/L), median (25th–75th percentile)	38,469 (35916–41,133)	41,153 (39844–43,773)	**0.001**
Large HDL particles (nmol/L), mean (± SD)	10,008 (3269)	11,229 (3811)	0.156
Small HDL particles (nmol/L), mean (± SD)	29,321 (5633)	31,128 (4895)	0.148
LDL particles (nmol/L), mean (± SD)	1,509 (369)	1,395 (301)	0.152
Large LDL particles (nmol/L), mean (± SD)	1,090 (279)	1,071 (249)	0.761
Small LDL particles (nmol/L), median (25th–75th percentile)	417 (302–568)	353 (244–454)	0.083
VLDL particles (nmol/L), median (25th–75th percentile)	3.5 (1.9–5.8)	2.9 (1.9–4.8)	0.659
Triglycerides/HDL ratio, median (25th–75th percentile)	1.11 (0.84–2.54)	0.86 (0.60–1.41)	**0.013**
Atherogenic coefficient, median (25th–75th percentile)	1.93 (1.43–2.59)	1.66 (1.21–2.10)	0.096
Atherogenic index, median (25th–75th percentile)	−0.32 (−0.44–0.04)	−0.43 (−0.58 to −0.21)	**0.012**

HDL, high-density lipoprotein; IDL, intermediate-density lipoprotein; LDL, low-density lipoprotein; TC, total cholesterol; VLDL, very-low-density lipoprotein. Data are presented as mean ± standard deviation or median (25th–75th percentile). TC, HDL, IDL, LDL, VLDL and triglycerides are expressed in mg/dL; HDL, LDL and VLDL particle concentrations in nmol/L; HDL, LDL and VLDL size in nm; triglycerides/HDL ratio is unitless; atherogenic coefficient is calculated as (TC–HDL)/HDL; atherogenic index as log10(triglycerides/HDL-cholesterol). All significant values are indicated in bold.

### 3.2 Correlation of lipid parameters with parameters of endothelial dysfunction within patients with lcSSc

CD31/CD42b– EMP correlated significantly with large LDL particles (*r* = 0.464, *p* = 0.044), while SDMA was significantly negatively correlated with VLDL size (*r* = −0.480, *p* = 0.033). In addition, positive but non-significant correlations were observed between CD31+/CD42b– EMP with IDL (*r* = 0.446, *p* = 0.066) and with VLDL (*r* = 0.443, *p* = 0.077). No other investigated parameter of endothelial dysfunction revealed significant correlations to lipid parameters (Table 3).

**Table 3:** Exploratory correlations of lipid parameters with parameters of endothelial dysfunction in patients with lcSSc.

		TC	HDL	IDL	LDL	VLDL	Triglycerides	HDL size	LDL size	VLDL size	Small HDL particles	Large HDL particles	HDL particles	Small LDL particles	Large LDL particles	LDL particles	VLDL particles	Triglycerides/HDL ratio	Atherogenic coeffeicient	Atherogenic index
FMD	*r*	−0.006	−0.306	0.106	0.127	0.001	−0.074	−0.240	−0.027	−0.319	0.132	−0.384	0.086	−0.072	0.053	0.075	−0.147	0.032	0.216	0.020
	*p*	>0.999	0.682	>0.999	>0.999	>0.999	>0.999	>0.999	>0.999	0.642	>0.999	0.275	>0.999	>0.999	>0.999	>0.999	>0.999	>0.999	>0.999	>0.999
NMD	*r*	−0.055	0.028	−0.174	−0.116	−0.157	−0.116	0.131	0.167	−0.223	−0.312	0.050	−0.242	−0.078	0.027	−0.142	−0.317	−0.058	−0.100	−0.069
	*p*	>0.999	>0.999	>0.999	>0.999	>0.999	>0.999	>0.999	>0.999	>0.999	0.957	>0.999	>0.999	>0.999	>0.999	>0.999	>0.999	>0.999	>0.999	>0.999
PWV	*r*	0.374	0.213	0.360	0.169	0.446	0.363	0.071	0.189	0.003	0.109	0.169	0.237	0.357	0.353	0.351	0.026	0.206	−0.006	0.217
	*p*	0.231	0.199	0.341	0.310	0.066	0.330	0.680	0.270	0.987	0.527	0.340	0.164	0.363	0.385	0.396	0.899	>0.999	>0.999	>0.999
Aix	*r*	0.381	0.014	0.360	0.329	0.276	0.262	−0.047	0.187	−0.055	0.058	0.034	0.031	0.294	0.247	0.314	−0.078	0.237	0.287	0.238
	*p*	0.198	0.933	0.341	0.484	0.103	0.112	0.786	0.276	0.750	0.736	0.848	0.858	0.097	0.146	0.682	0.705	>0.999	0.880	>0.999
CD31+/CD42b− EMP	*r*	0.395	−0.015	0.446	0.387	0.443	0.304	−0.100	0.222	−0.405	0.040	−0.187	0.044	0.087	**0.464**	0.367	0.259	0.254	0.294	0.255
	*p*	0.154	0.929	0.066	0.176	0.077	0.704	0.561	0.192	0.154	0.816	0.290	0.798	0.630	**0.044**	0.308	0.201	>0.999	0.803	>0.999
ADMA	*r*	0.187	0.009	0.218	0.304	0.073	−0.055	−0.005	0.100	−0.280	−0.171	−0.123	−0.206	0.355	0.237	0.267	−0.216	−0.058	0.146	−0.057
	*p*	0.261	0.955	0.201	0.693	0.672	0.743	0.975	0.564	0.098	0.320	0.489	0.229	0.462	0.165	0.115	0.288	>0.999	>0.999	>0.999
SDMA	*r*	0.084	0.066	0.205	0.084	0.147	−0.003	−0.020	0.056	**−0.480**	−0.154	−0.038	−0.105	0.181	0.227	0.191	−0.129	−0.026	−0.004	−0.025
	*p*	0.617	0.692	0.229	0.618	0.393	0.987	0.909	0.747	**0.033**	0.368	0.831	0.542	0.315	0.183	0.265	0.529	>0.999	>0.999	>0.999
Arginine	*r*	−0.164	0.072	−0.289	−0.226	−0.208	−0.213	0.124	−0.117	−0.205	−0.158	0.087	−0.038	−0.087	−0.027	−0.103	−0.205	−0.219	−0.161	−0.213
	*p*	0.325	0.669	0.957	0.172	0.223	0.199	0.472	0.497	0.229	0.358	0.625	0.825	0.631	0.874	0.549	0.315	>0.999	>0.999	>0.999
Homoarginine	*r*	−0.115	−0.107	−0.141	−0.085	−0.053	0.140	0.101	−0.034	0.220	−0.114	−0.003	−0.070	−0.284	−0.183	−0.207	0.189	0.146	0.107	0.148
	*p*	>0.999	>0.999	>0.999	>0.999	>0.999	>0.999	>0.999	>0.999	>0.999	>0.999	>0.999	>0.999	>0.999	>0.999	>0.999	>0.999	>0.999	>0.999	>0.999
vWF: Ac	*r*	0.108	0.027	0.051	0.008	0.020	0.043	0.074	0.053	−0.086	−0.024	−0.047	0.053	0.184	0.087	0.082	−0.213	0.006	−0.037	0.014
	*p*	>0.999	>0.999	>0.999	>0.999	>0.999	>0.999	>0.999	>0.999	>0.999	>0.999	>0.999	>0.999	>0.999	>0.999	>0.999	>0.999	>0.999	>0.999	>0.999
vWF: Ag	*r*	0.198	0.139	0.108	0.043	0.054	0.108	0.151	0.082	−0.049	−0.062	0.052	0.044	0.277	0.153	0.151	−0.221	−0.003	−0.067	0.008
	*p*	>0.999	>0.999	>0.999	>0.999	>0.999	>0.999	>0.999	>0.999	>0.999	>0.999	>0.999	>0.999	>0.999	>0.999	>0.999	>0.999	>0.999	>0.999	>0.999

ADMA, asymmetric dimethylarginine; Aix, augmentation index; EMP, endothelial microparticles; FMD, flow-mediated dilation; NMD, nitroglycerin-mediated dilation; PWV, pulse-wave velocity; vWF: Ac, von Willebrand factor activity; vWF: Ag, von Willebrand factor antigen; TC, total cholesterol; HDL, high-density lipoprotein; IDL, intermediate-density lipoprotein; LDL, low-density lipoprotein; VLDL, very low-density lipoprotein. Lipid units are as in Table 2. All significant values are indicated in bold.

### 3.3 Correlation of lipid parameters with lcSSc-specific parameters

No statistically significant correlations were detected between lipid parameters and lcSSc-specific parameters. There was only a positive, but non-significant correlation between mRSS with small HDL particles (*r* = 0.413, *p* = 0.096) and a negative, non-significant correlation between HDL and DETECT score step 1 points (*r* = −0.430, *p* = 0.096) (Supplementary Table 1).

### 3.4 Association of lipid parameters with vascular and clinical events in lcSSc

ANOVA revealed significant associations between macrovascular events and small LDL particles and triglycerides/HDL ratio (*η*^2^ = 0.277, *p* = 0.018; *η*^2^ = 0.217, *p* = 0.036, respectively) as well as between ILD, small LDL particles, LDL particles, triglycerides/HDL ratio and atherogenic coefficient (*η*^2^ = 0.266, *p* = 0.024; *η*^2^ = 0.340, *p* = 0.003; *η*^2^ = 0.221, *p* = 0.036; *η*^2^ = 0.272, *p* = 0.012, respectively). No further associations between outcome parameters and lipid parameters were observed (Table 4).

**Table 4:** Exploratory associations of lipid parameters with vascular and clinical events in lcSSc.

		DU	PAH	Microvascular event	Macrovascular event	ILD	Clinical event
TC	*η* ^2^	0.059	0.050	0.043	0.031	0.136	0.115
	*p*	>0.999	>0.999	>0.999	>0.999	0.210	0.390
HDL	*η* ^2^	0.000	0.005	0.012	0.077	0.069	0.082
	*p*	>0.999	>0.999	>0.999	0.714	0.840	>0.999
IDL	*η* ^2^	0.074	0.032	0.056	0.018	0.150	0.146
	*p*	0.798	>0.999	>0.999	>0.999	0.168	0.192
LDL	*η* ^2^	0.063	0.030	0.067	0.056	0.192	0.171
	*p*	0.948	>0.999	0.876	>0.999	0.066	0.156
VLDL	*η* ^2^	0.021	0.061	0.017	0.055	0.188	0.100
	*p*	>0.999	>0.999	>0.999	>0.999	0.078	0.888
Triglycerides	*η* ^2^	0.015	0.031	0.028	0.098	0.149	0.128
	*p*	>0.999	>0.999	>0.999	0.456	0.162	>0.999
HDL size	*η* ^2^	0.005	0.001	0.049	0148	0.102	0.169
	*p*	>0.999	>0.999	>0.999	0.180	0.450	>0.999
LDL size	*η* ^2^	0.033	0.185	0.007	0.056	0.013	0.072
	*p*	>0.999	0.084	>0.999	>0.999	>0.999	>0.999
VLDL size	*η* ^2^	0.009	0.001	0.022	0.036	0.003	0.040
	*p*	>0.999	>0.999	>0.999	>0.999	>0.999	>0.999
Small HDL particles	*η* ^2^	0.009	0.004	0.035	0.038	0.005	0.049
	*p*	>0.999	>0.999	>0.999	>0.999	>0.999	>0.999
Large HDL particles	*η* ^2^	0.000	0.000	0.000	0.000	0.014	0.004
	*p*	>0.999	>0.999	>0.999	>0.999	>0.999	>0.999
HDL particles	*η* ^2^	0.001	0.006	0.002	0.001	0.020	0.001
	*p*	>0.999	>0.999	>0.999	>0.999	>0.999	>0.999
Small LDL particles	*η* ^2^	0.018	0.021	0.147	**0.277**	**0.266**	0.385
	*p*	>0.999	>0.999	0.240	**0.018**	**0.024**	0.090
Large LDL particles	*η* ^2^	0.006	0.043	0.003	0.027	0.182	0.057
	*p*	>0.999	>0.999	>0.999	>0.999	0.090	>0.999
LDL particles	*η* ^2^	0.028	0.006	0.074	0.147	**0.340**	0.261
	*p*	>0.999	>0.999	0.798	0.180	**0.003**	0.096
VLDL particles	*η* ^2^	0.003	0.042	0.011	0.115	0.057	0.115
	*p*	>0.999	>0.999	>0.999	0.678	>0.999	>0.999
Triglycerides/HDL ratio	*η* ^2^	0.005	0.020	0.035	**0.217**	**0.221**	0.236
	*p*	>0.999	>0.999	>0.999	**0.036**	**0.036**	0.960
Atherogenic coefficient	*η* ^2^	0.015	0.016	0.055	0.203	**0.272**	0.252
	*p*	>0.999	>0.999	>0.999	0.054	**0.012**	0.372
Atherogenic index	*η* ^2^	0.022	0.022	0.043	0.113	0.151	0.153
	*p*	>0.999	>0.999	>0.999	0.330	0.130	0.714

DU, digital ulcers; ILD, interstitial lung disease; PAH, pulmonary arterial hypertension; TC, total cholesterol; HDL, high-density lipoprotein; IDL, intermediate-density lipoprotein; LDL, low-density lipoprotein; VLDL, very low-density lipoprotein. Lipid units and derived indices (triglycerides/HDL ratio, atherogenic coefficient, atherogenic index) are defined as in Table 2. All significant values are indicated in bold.

## 4 Discussion

Our findings demonstrate that patients with lcSSc exhibit dyslipidemia, characterized by significantly lower HDL and total HDL particles as well as elevated triglyceride levels. These findings underscore the need for clinicians to consider extended lipid profiling beyond conventional lipid panels. On the other hand, however, the observed group differences and event-related associations were of modest magnitude, and most lipid parameters did not differ significantly, particularly after multiple testing. Borderline or isolated associations, especially those that did not remain robust after correction for multiple comparisons, should therefore be regarded as exploratory signals rather than established effects. Given the small sample size and exploratory design of this study, it must be noted that these observations should be interpreted as descriptive and hypothesis-generating and requires confirmation in larger studies. Accordingly, the applied Holm–Bonferroni correction reduces the risk of Type I error but, in this small cohort, also increases the probability of Type II error, so non-significant results should not be overinterpreted as evidence of absence of association. The present work should therefore be interpreted as a complementary biomarker-focused sub-study expanding previous EFVELSS analyses rather than an independent cohort investigation.

HDL particle number reduction suggests altered lipid metabolism, but it does not necessarily equate to impaired HDL functionality. HDL particles are heterogeneous with various protective biological functions, including cholesterol efflux capacity, antioxidative and immunomodulatory effects, which are not directly assessed in this study (29, 30). Consistent with robust evidence from large population-based cohorts and meta-analyses, reduced HDL particle number and concentration are independently associated with an increased risk of atherosclerotic cardiovascular disease, beyond traditional HDL cholesterol measurements (5, 29–31). Specifically, each standard deviation increase in HDL particle concentration corresponds to a significant reduction in cardiovascular events and mortality, underscoring the superior predictive value of HDL particle metrics over conventional HDL levels. Nevertheless, the functional quality of HDL particles cannot be inferred from particle number alone. Therefore, direct mechanistic assessments including cholesterol efflux assays remain essential to confirm HDL dysfunction and to elucidate its contribution to disease pathogenesis in patients with lcSSc. Evaluation of quantitative and functional lipoprotein parameters in clinical practice may improve risk stratification in lcSSc management and are thus needed. In line with this, the present data do not allow for conclusions about HDL function or causality rather to point out lipid parameters that merit more detailed functional evaluation in future studies. Our observational data provide preliminary evidence that lipid abnormalities are present in early-stage lcSSc and may be relevant for disease assessment in this population. The observed dyslipidemic pattern is further reflected by an increased triglycerides/HDL ratio and atherogenic index, both of which integrate HDL and triglycerides as subcomponents. The triglycerides/HDL ratio serves as a predictor of cardiovascular risk with a normal value <3.5 mg/dL, while the atherogenic index reflects the balance between protective and atherogenic lipoproteins, with values >0.24 considered high risk (32, 33). In our cohort with lcSSc, both parameters were significantly elevated compared to controls, underscoring their utility as surrogate markers of vascular risk, although both parameters were within normal ranges compared to published reference values (1–4, 7, 9, 32–34). This pattern suggests a potentially less favorable vascular risk profile in lcSSc, but the magnitude of these differences is modest and should not be overinterpreted as clinically established risk in the absence of longitudinal outcome data. Dyslipidemia, particularly the combination of low HDL and high triglycerides, has been associated with subclinical atherosclerosis and increased carotid intima-media thickness, further linking lipid abnormalities to vascular risk in SSc. Similar patterns of inflammation-driven subclinical atherosclerosis have been reported in other inflammatory rheumatic diseases, such as psoriatic arthritis, where increased carotid intima-media thickness has been observed even in patients without traditional cardiovascular risk factors (9, 34). Proposed mechanistic contributors include anti-lipoprotein lipase antibodies that inhibit enzymatic triglyceride clearance and impaired cholesterol efflux affecting HDL function, which was, however, not measured in our study (5, 7). In the current work, these mechanisms are mentioned only to contextualize our findings within the existing literature and remain speculative.

Our analysis highlights a potential interplay between lipoprotein metabolism and endothelial dysfunction, a recognized contributor to vascular injury in lcSSc, although our results revealed only sporadic correlations. In our cohort with lcSSc, circulating EMPs correlated positively with large LDL particle counts, suggesting a potential role of specific LDL subfractions in endothelial activation. Large LDL particles possess pro-atherogenic and pro-inflammatory properties, which may promote EMP release, reflecting ongoing microvascular injury and vascular involvement in early lcSSc (11, 35, 36). Additionally, the negative correlation between SDMA levels and VLDL size may indicate a link between endothelial dysfunction and lipoprotein composition rather than total VLDL particle burden. SDMA-mediated inhibition of endothelial nitric oxide (NO) synthase reduces NO bioavailability, contributing to vascular stiffness and microvascular damage (11, 37, 38). These findings suggest that LDL-driven endothelial activation and SDMA-associated NO impairment may be potentially involved in microvascular dysfunction in lcSSc. Nevertheless, due to the small sample size with an exploratory design, these correlations should be regarded as descriptive and hypothesis-generating. Future studies with larger populations are necessary to validate these findings.

Although LDL subfractions have been implicated in cardiovascular risk in SSc and proprotein convertase subtilisin/kexin 9 have been associated with disease activity in diffuse cutaneous SSc, our analysis did not reveal significant correlations between any investigated lipid parameter and clinical or disease activity parameters of lcSSc (39, 40). This lack of correlation may be reflected by the very small sample size and cohort characteristics, but also reflects the heterogeneity of SSc and potentially the early stage of lcSSc in our cohort. Furthermore, lipid-related changes in SSc are closely tied to inflammatory processes and immune dysregulation, which might be less pronounced in our cohort with early-stage lcSSc. Taken together, the predominantly null findings regarding clinical correlations emphasize that the current data does not support lipid markers as established disease activity biomarkers in lcSSc and that larger studies are needed.

Small and total LDL particles were significantly associated with ILD development in our cohort, which is consistent with prior multi-omic study linking elevated LDL and triglycerides to ILD progression and fibrogenesis (8). In addition, triglycerides/HDL ratio and atherogenic coefficient were also associated with ILD, reflecting a shift toward a more atherogenic lipid profile that may promote fibrotic remodeling (41). Importantly, the number of macrovascular and ILD events was small, so these ANOVA-derived associations must be interpreted with particular caution and considered hypothesis-generating. Elevated LDL and triglycerides may promote fibroblast activation and tissue injury via oxidative stress, pro-inflammatory cytokine release, and endothelial dysfunction. Lipoprotein alterations could enhance transforming growth factor *β* signaling and endothelial-to-mesenchymal transition, pivotal processes in fibrosis progression. This is supported by translational studies showing disrupted LDL receptor pathways and increased LDL-mediated apoptosis in fibroblast and endothelial cells within models of pulmonary fibrosis (42–44). Triglycerides may further promote fibrosis via anti-lipoprotein lipase antibodies, impairing triglyceride clearance and increasing substrate availability for fibroblast proliferation and extracellular matrix deposition (7, 8, 45). These mechanistic insights highlight the potential of lipid metabolism modulation as a therapeutic target in lcSSc-related ILD. Nevertheless, since larger prospective or therapeutic studies evaluating the clinical influence of lipid modification in ILD are missing, these associations should be interpreted cautiously.

Overall, our findings suggest that lipid abnormalities in lcSSc may arise from several, partly overlapping processes. Some associations, such as those with ILD and macrovascular events, may reflect a closer link between lipid subfractions and lcSSc-related disease activity or progression. In contrast, other alterations are likely secondary to systemic inflammation, endothelial activation, or concomitant treatment, rather than primary drivers of disease. Given the observational design, our data do not allow separation of these pathways, and causal inferences should be avoided.

Strengths of this study are a well-defined cohort of patients with early-stage lcSSc that reduces confounding from advanced vascular complications, a control cohort at baseline and the use of detailed lipoprotein profiling combined with endothelial biomarkers, which allow a comprehensive, albeit exploratory, description of lipid and endothelial parameters in this population. Limitations are the small sample size and single-center design limiting statistical power and generalizability. The ANOVA-based associations are based on a low number of macrovascular and ILD events, which limits statistical power and precision of the effect size estimates and increases the risk of chance findings. Furthermore, the cohort exclusively comprised patients with lcSSc, so the present results should not be directly extrapolated to diffuse cutaneous SSc or other SSc subsets. The observational design precludes causal inference, and direct assessments of lipoprotein functionality, such as cholesterol efflux or antioxidative assays, were not performed. Other advanced lipidomic or proteomic analyses were also beyond this study’s scope. Multiple testing adjustments may have reduced significance for some associations, underscoring the risk of Type II error on the one hand, but on the other hand, it minimizes incidental significant associations. Finally, potential confounding variables known to influence lipid markers like dietary habits, and interpreting reduced HDL particle numbers as impaired functionality are further limitations. Overall, these limitations support a cautious interpretation of the data and argue against drawing firm mechanistic or clinical conclusions from this single exploratory study.

In conclusion, patients with lcSSc exhibit alterations in lipid metabolism characterized by significantly lower HDL and total HDL particles as well as elevated triglycerides compared to controls, but with scarce findings for other lipid measures and clinical correlations. This dyslipidemic profile may be associated with disease complications in lcSSc. However, given the small sample size, exploratory design and lack of multivariable adjustment, these associations should be interpreted as preliminary and hypothesis-generating. Further studies with larger and more diverse cohorts, including patients with diffuse cutaneous SSc, and mechanistic analyses are needed to clarify these associations and their relevance across the SSc spectrum.

## Data Availability

The raw data supporting the conclusions of this article will be made available by the authors, without undue reservation.
