# Supplementary material for: Association of lipid parameters with the development of disease complications in patients with limited cutaneous systemic sclerosis: a prospective exploratory cohort study
Source: Front Nutr. 2026 Mar 24;13:1794319. doi: 10.3389/fnut.2026.1794319 (PMC13053233; doi:10.3389/fnut.2026.1794319)
Supplement: Supplementary file 1 [file Table_1.DOCX]

Supplementary Material

**Supplementary Table 1.** Correlation analysis of lipid parameters with clinical parameters at baseline within patients with lcSSc adjusted for multiple testing.

|  | | **TC** | **HDL** | **IDL** | **LDL** | **VLDL** | **Triglycerides** | **HDL size** | **LDL size** | **VLDL size** | **Small HDL particles** | **Large HDL particles** | **HDL particles** | **Small LDL particles** | **Large LDL particles** | **LDL partciles** | **VLDL particles** | **Triglycerides/HDL ratio** | **Atherogenic coeffeicient** | **Atherogenic index** |
| --- | --- | --- | --- | --- | --- | --- | --- | --- | --- | --- | --- | --- | --- | --- | --- | --- | --- | --- | --- | --- |
| **CSURI** | r | 0.216 | -0.065 | 0.185 | 0.125 | 0.290 | 0.249 | -0.120 | 0.037 | 0.055 | 0.018 | -0.243 | 0.095 | 0.361 | 0.003 | 0.085 | -0.013 | 0.154 | 0.118 | 0.160 |
|  | p | >0.999 | >0.999 | >0.999 | >0.999 | 0.688 | >0.999 | >0.999 | >0.999 | >0.999 | >0.999 | >0.999 | >0.999 | 0.312 | >0.999 | >0.999 | >0.999 | >0.999 | >0.999 | >0.999 |
| **MES** | r | 0.101 | 0.058 | 0.071 | 0.077 | 0.081 | 0.044 | 0.055 | 0.045 | 0.221 | -0.103 | 0.179 | -0.098 | 0.104 | 0.026 | 0.078 | -0.075 | 0.004 | 0.036 | 0.008 |
|  | p | >0.999 | >0.999 | >0.999 | >0.999 | >0.999 | >0.999 | >0.999 | >0.999 | >0.999 | >0.999 | >0.999 | >0.999 | >0.999 | >0.999 | >0.999 | >0.999 | >0.999 | >0.999 | >0.999 |
| **mRSS** | r | 0.131 | -0.212 | 0.186 | 0.181 | 0.299 | 0.371 | -0.363 | -0.053 | 0.300 | 0.413 | -0.266 | 0.223 | 0.299 | -0.053 | 0.192 | 0.364 | 0.355 | 0.328 | 0.357 |
|  | p | >0.999 | >0.999 | >0.999 | >0.999 | 0.608 | 0.176 | 0.304 | >0.999 | 0.600 | 0.096 | >0.999 | >0.999 | 0.728 | >0.999 | >0.999 | 0.544 | 0.232 | 0.360 | 0.224 |
| **UCLA SCTC GIT 2.0 total score** | r | -0.046 | 0.015 | -0.081 | 0.052 | -0.184 | -0.308 | -0.012 | -0.065 | 0.197 | 0.127 | 0.047 | -0.019 | 0.056 | -0.070 | 0.068 | -0.111 | -0.233 | 0.033 | -0.230 |
|  | p | >0.999 | >0.999 | >0.999 | >0.999 | >0.999 | 0.480 | >0.999 | >0.999 | >0.999 | >0.999 | >0.999 | >0.999 | >0.999 | >0.999 | >0.999 | >0.999 | >0.999 | >0.999 | >0.999 |
| **UCLA SCTC GIT constipation score** | r | -0.100 | -0.061 | -0.096 | -0.068 | -0.163 | -0.242 | -0.014 | -0.109 | 0.088 | 0.079 | -0.042 | 0.113 | -0.155 | -0.093 | -0.079 | 0.194 | -0.205 | -0.015 | -0.206 |
|  | p | >0.999 | >0.999 | >0.999 | >0.999 | >0.999 | >0.999 | >0.999 | >0.999 | >0.999 | >0.999 | >0.999 | >0.999 | >0.999 | >0.999 | >0.999 | >0.999 | >0.999 | >0.999 | >0.999 |
| **DETECT score step 1** | r | 0.042 | -0.430 | 0.091 | 0.058 | 0.244 | 0.226 | -0.324 | -0.121 | -0.056 | -0.019 | -0.296 | -0.262 | 0.229 | 0.050 | 0.042 | -0.430 | .0217 | 0.145 | 0.219 |
|  | p | >0.999 | 0.096 | >0.999 | >0.999 | >0.999 | >0.999 | 0.432 | >0.999 | >0.999 | >0.999 | 0.896 | >0.999 | >0.999 | >0.999 | >0.999 | 0.096 | >0.999 | >0.999 | >0.999 |
| **DETECT score step 2** | r | 0.089 | -0.360 | 0.129 | 0.103 | 0.249 | 0.204 | -0.292 | -0.084 | -0.086 | -0.048 | -0.195 | -0.241 | 0.217 | 0.077 | 0.089 | -0.360 | 0.203 | -0.010 | 0.205 |
|  | p | >0.999 | 0.312 | >0.999 | >0.999 | >0.999 | >0.999 | >0.999 | >0.999 | >0.999 | >0.999 | >0.999 | >0.999 | >0.999 | >0.999 | >0.999 | 0.312 | >0.999 | >0.999 | >0.999 |
| **EUSTAR index** | r | -0.028 | -0.249 | -0.014 | 0.004 | 0.096 | 0.230 | -0.298 | -0.173 | 0.393 | 0.343 | -0.318 | 0.219 | 0.266 | -0.278 | -0.027 | 0.056 | 0.201 | 0.212 | 0.205 |
|  | p | >0.999 | >0.999 | >0.999 | >0.999 | >0.999 | >0.999 | 0.896 | >0.999 | 0.144 | 0.328 | 0.536 | >0.999 | >0.999 | 0.808 | >0.999 | >0.999 | >0.999 | >0.999 | >0.999 |

Abbreviations: CSURI: capillaroscopic skin ulcer risk index; HDL: high-density lipoprotein; IDL: intermediate-density lipoprotein; LDL: low-density lipoprotein; MES: microangiopathy evolution score; mRSS: modified Rodnan Skin Score; TC: total cholesterol; VLDL: very-low-density lipoprotein
